# 

**Published:** 1855-10

**Authors:** 


*To Subscribers.*—The delay in mailing of the last (September) number of the *Journal*, was occasioned by the necessity of enclosing *bills* to those subscribers who stand in arrears on our books. To fill out and enclose nearly *one thousand bills*, is a task requiring both time and patience ; and we had hoped that we should be relieved from the task by the prompt payment of subscriptions by our readers—but we have been disappointed to a very great extent. It requires only two more numbers to complete the volume and the subscription year. Will our subscribers act promptly? They need have no fears of sending through the Post-Office, if they will be particular to address their letters to N. S. DAVIS, Chicago, Illinois.

